# Multimodal Brain Tumor Classification Using Deep Learning and Robust Feature Selection: A Machine Learning Application for Radiologists

**DOI:** 10.3390/diagnostics10080565

**Published:** 2020-08-06

**Authors:** Muhammad Attique Khan, Imran Ashraf, Majed Alhaisoni, Robertas Damaševičius, Rafal Scherer, Amjad Rehman, Syed Ahmad Chan Bukhari

**Affiliations:** 1 Department of Computer Science, HITEC University, Museum Road, Taxila 47080, Pakistan; attique.khan440@gmail.com; 2 Department of Computer Engineering, HITEC University, Museum Road, Taxila 47080, Pakistan; imran.ashraf@hitecuni.edu.pk; 3 College of Computer Science and Engineering, University of Ha’il, Ha’il 81451, Saudi Arabia; m.alhaisoni@uoh.edu.sa; 4 Faculty of Applied Mathematics, Silesian University of Technology, 44-100 Gliwice, Poland; 5 Department of Applied Informatics, Vytautas Magnus University, 44404 Kaunas, Lithuania; 6 Department of Intelligent Computer Systems, Czestochowa University of Technology, 42-200 Czestochowa, Poland; rafal.scherer@pcz.pl; 7 College of Computer and Information Sciences, Prince Sultan University, Riyadh 11586, Saudi Arabia; rkamjad@gmail.com; 8 Division of Computer Science, Mathematics and Science, Collins College of Professional Studies, St. John’s University, New York, NY 11439, USA; bukharis@stjohns.edu

**Keywords:** brain tumor, healthcare, linear contrast, transfer learning, deep learning features, feature selection, feature fusion, PLS, ELM

## Abstract

Manual identification of brain tumors is an error-prone and tedious process for radiologists; therefore, it is crucial to adopt an automated system. The binary classification process, such as malignant or benign is relatively trivial; whereas, the multimodal brain tumors classification (T1, T2, T1CE, and Flair) is a challenging task for radiologists. Here, we present an automated multimodal classification method using deep learning for brain tumor type classification. The proposed method consists of five core steps. In the first step, the linear contrast stretching is employed using edge-based histogram equalization and discrete cosine transform (DCT). In the second step, deep learning feature extraction is performed. By utilizing transfer learning, two pre-trained convolutional neural network (CNN) models, namely VGG16 and VGG19, were used for feature extraction. In the third step, a correntropy-based joint learning approach was implemented along with the extreme learning machine (ELM) for the selection of best features. In the fourth step, the partial least square (PLS)-based robust covariant features were fused in one matrix. The combined matrix was fed to ELM for final classification. The proposed method was validated on the BraTS datasets and an accuracy of 97.8%, 96.9%, 92.5% for BraTs2015, BraTs2017, and BraTs2018, respectively, was achieved.

## 1. Introduction

A brain tumor is an abnormal growth of brain cells in an uncontrollable way [1,2]. Brain tumors can be cancerous or noncancerous. The gravity inside the skull can accelerate the growth of a brain tumor. In the worst case, it can cause brain damage, which can be life-threatening. According to an estimate, 18,020 adults will die from the primary cancerous brain and central nervous system (CNS) tumors in 2020 [3]. Various manifestations and classes of brain tumors have different appearances on magnetic resonance imaging (MRI) data [4,5]. Therefore, MRI scans are typically used to detect and classify brain tumors. MRI assists doctors in evaluating tumors in order to plan for further treatment. This treatment depends on various factors like shape, size, type, grade, and location of cancer. Depending on the patient’s condition, these factors can have enormous variation. Hence, accurate recognition and classification of brain tumors are critical for proper treatment [6].

Manual identification of brain tumors and tracking their changes over time are tedious and error-prone activities [7]; hence, automated systems are required to replace the conventional manual methods. In the last decade, the results of deep neural networks (DNNs) exhibited excellent performance, which is also evident from the recent multimodal BraTS challenges [8]. Another well-known technique of deep learning is convolutional neural networks (CNN), which shows excellent performance both for 2D and 3D medical images [9,10]. Similarly, the transfer learning technique is typically utilized in case of limited availability of data and computational resources to save time [11]. This technique uses the knowledge acquired for one task to solve related ones [12]. Feature fusion is the detection of co-related features in order to fuse them to identify and compact a set of salient features to improve the detection accuracy. Furthermore, to reduce the time and space complexity, intelligent feature selection is typically required [13,14].

### 1.1. Significant Challenges and Motivation

**Figure 1:**
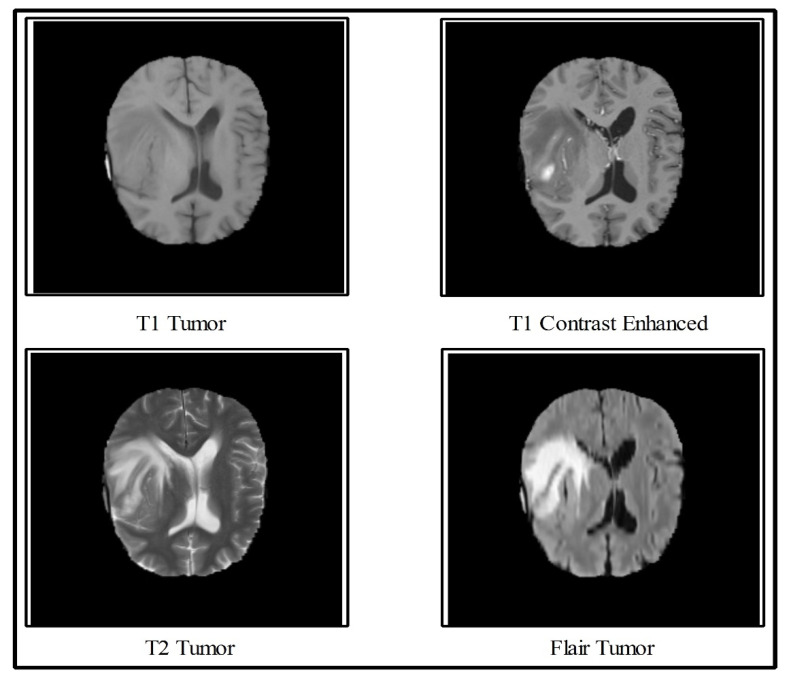
Sample images collected from Multimodal Brain Dataset (BRATS) [15].

In the multimodal brain tumor classification task, several challenges exist, which reduce automated system performance. In the classification task, two steps are usually performed—features extraction and classifiers-based classification. The features extraction is an essential step in pattern recognition, which predicts an object based on its crucial characteristics like shape, color, names, and a few more. However, the performance of classifiers depends on the strength of the extracted features. The recent success of deep learning in the medical domain reflects the interest of researchers of computer vision. However, all extracted deep learning features are normally not useful for correct classification and they might consume much time during the execution process. Moreover, the similarity among some tumors like T2 and Flair tumors is also very high, as demonstrated in Figure 1, which makes this classification process even more complicated. Further, the T1 tumors and T1 contrast-enhanced tumors have shallow contrast, which is another challenge for correct feature extraction.

In this work, we propose a deep learning scheme for multimodal brain tumors classification. To handle the problem of shallow contrast, we implemented a linear contrast enhancement technique, which was further refined through histogram equalization. Transfer learning was used for feature extraction from two different CNN models and the fusion was performed. The motivation behind the fusion of two CNN models was to get a new feature vector with more information. Although this process improved accuracy, the computational time was affected. To further enhance efficiency and computational time, we proposed a feature selection technique. The robust features obtained using this technique were later classified through the Extreme Learning Machine (ELM).

### 1.2. Major Contributions

- We divided the image into two clusters based on a K-Means clustering algorithm and applied edge-based histogram equalization on each image. Further, the discrete cosine transform (DCT) was utilized for local information enhancement.
- Deep learning features were extracted from two pre-trained CNN models through transfer learning (TL). The last FC layer was used in both models for feature extraction.
- The Partial Least Square (PLS) based features of both CNN models were fused in one matrix.
- The robust features were selected using correntropy-based joint group learning. The robust features were finally classified using the ELM classifier.
- Three datasets such as BRATS 2015, BRATS 2017, and BRATS 2018 were used for the experiments and the statistical analysis to examine the scalability of the proposed classification scheme.

## 2. Related Work

Classification of multimodal brain tumors (i.e., T1, T2, T1CE, and Flair) required the determination of altered features, such as shape and texture in the MRI Image [16]. The popular method of diagnosis of these tumors—which spread widely among computer vision researchers—is a computer-aided diagnosis (CAD) system [1,17]. In a CAD system, two main stages are involved—first, tumor preprocessing and detection, and second, classifying the tumor into a relevant category. In this work, we focused on the classification task of multimodal brain tumors. For classification, we used the BRATS series based on few top submissions [18,19,20,21]. Amin et al. [22] introduced a CNN framework for brain tumor classification. In the presented method, the DWT fusion process was performed to improve the original MRI scan and then a partial diffusion filter was employed for noise removal. Later on, they used a global thresholding algorithm for tumor extraction that passed to the CNN model for classification of tumors into the related categories. Five BRATS datasets, namely, BRATS2012, 2013, 2015, 2018, and BRATS2013 were used and showed improved performance on the fusion approach. Sajjad et al. [23] presented a CNN-based multimodal tumor classification system. They initially segmented the tumor regions in the MRI scans using CNN. Then, they performed an extensive data augmentation to train a good CNN model. Later on, they fine-tuned the pre-trained CNN model using augmented brain data. The last layer was used as a classification of tumors in the presented method and it showed that augmented data gave better results on the selected datasets.

Sharif et al. [24] presented an active deep learning system for the segmentation and classification of brain tumors. They initially performed contrast enhancement, and the resultant image was passed to the Saliency-based Deep Learning (SbDL) method, for the construction of a saliency map. The thresholding was applied in the next step, and the resultant images were used to fine-tune the pre-trained CNN model Inception V3. Further, they also extracted dominant rotated local binary pattern (DRLBP) features, fused with CNN features. Later on, a PSO-based optimization was performed and the optimal vector was passed to the Softmax classifier for final classification. They used BRATS 2015, 2017, and 2018 datasets for evaluation, and achieved improved classification accuracy. In [25], the authors presented a CNN-based scheme for the classification of brain tumors. They considered the problem of structural variability of the tumor around the adjacent regions. For this purpose, they designed small kernels to keep the weights of each neuron very small. Taking advantage of these weights, they achieved an accuracy of 97.5%.

Vijh et al. [26] presented an adaptive particle swarm optimization (PSO) with the Otsu method to find the optimal threshold value. Later, they applied anisotropic diffusion (AD) filtering on brain MRI images to cancel noise and improve image quality. Features were extracted from enhanced images that were used both for training the CNN and performing the classification. Other methods were also introduced in the literature for brain tumor classification, such as a generative adversarial network (GAN)-based approach [19], artificial neural network (ANN)-based learning [27], ELM-based learning [28], residual network [29], standard-features-based classification [30,31], adaptive independent subspace analysis [32], transfer learning-based tumors classification [33], and Excitation DNN [34]. In addition, Toğaçar et al. [35] proposed a hybrid method based on CNN and feature selection, for the classification of brain tumors. They achieved an improved accuracy of above 90%. In the above techniques, they did not provide the computational time. However, the computational time was most needed for this current era for each automated system. The more recent, Muhammad et al. [36] presented a detailed review on multi-grade brain tumor classification. They presented a detailed description of brain tumor classification (BTC) steps like preprocessing of tumor, deep learning features, and classification. They discussed detailed limitations and achievements of existing deep learning techniques for BTC. In addition, they also presented the importance of transfer learning for deep learning feature extraction.

## 3. Proposed Methodology

**Figure 2:**
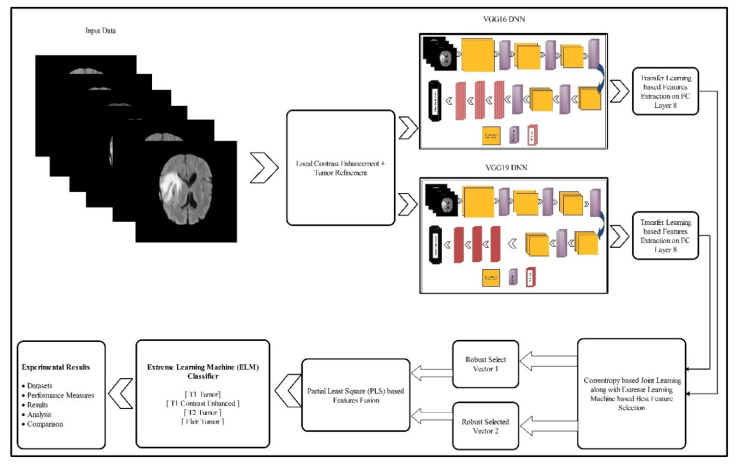
Proposed architecture for multimodal brain tumor classification using deep learning features and extreme learning machine (ELM).

In this section, the proposed methodology for multimodal brain tumor classification using deep learning is presented. The proposed method consists of five core steps—linear contrast stretching, deep learning features extraction using transfer learning, a correntropy-based joint learning approach along with ELM for best features selection, the PLS-based fusion of the selected features, and finally the ELM-based classification. The testing of the proposed method was performed on the BRATS datasets. The performance of the approach was checked using standard performance measures like accuracy and false negative rate (FNR). Furthermore, the performance of the proposed work was also reported by measuring the execution time. A detailed flow of the proposed methodology is illustrated in Figure 2. In the following, the technical description of each step is provided.

### 3.1. Linear Contrast Enhancement

Improving the graphic features of an image is the primary objective of contrast enhancement. It is a preprocessing step that is used in many applications like biomedical imaging, agriculture infections diagnosis, and some others [37,38,39,40,41,42]. The impact of low contrast images is not useful for feature extraction, as visually, tumors are not visible and error prone. Therefore, in this step, we improved the linear contrast of an image, which showed the main impact on the tumor region. For this purpose, we implemented a hybrid technique. In this technique, initially, we split the image into two parts using the K-Means clustering algorithm. Then, edge-based texture histogram equalization (HE) was applied. Later on, DCT was applied to combine both clusters in one image. The resulting image had enhanced contrast as compared to the original one. The mathematical formulation of this method is given as follows:

Consider, we have a dataset $\Delta =\left\{\tau_{1},\;\tau_{2},\;\tau_{3},\ldots ,\;\tau_{N}\right\}$, $\tau_{N}\in {\mathbb{R}}^{d}$. Consider τ(x,y) is an MRI image of dimension N×M where N=256 and M=256, rows, and columns, respectively. Let $\tau_{i}$ denotes the average of clusters $K_{i}$ then using this, the criterion function is defined as follows:

(1) $$\bar{S}=\sum_{i=1}^{K}\sum_{\tau \in K_{i}}{\left|\tau -\tau_{i}\right|}^{2},$$

where $\bar{S}$ denotes the sum of square error of all pixels, $\tau_{i}$ means input images, and K implies the number of clusters that are initialized in this work. In K-Means, the Euclidean distance was used to as criterion distance, which was defined as follows:

(2) $$D\left(\tau_{i},\;y_{i}\right)=\sqrt{\sum_{i=1}^{n}{\left(\tau_{i}-y_{i}\right)}^{2}},$$

where $\tau_{i}$ and $y_{i}$ are two vectors. This formulation obtained two clusters. Using resultant images defined by $\tau_{1}\left(x,y\right)$, we employed edge-based texture HE, where $\tau_{1}\left(x,y\right)\in \bar{S}$. For the resultant image $\tau_{1}\left(x,y\right)$, the gradient was computed as follows:

(3) $$G\left(x,y\right)=\sqrt{G_{x}{\left(x,y\right)}^{2}+G_{y}{\left(x,y\right)}^{2}},$$

where $G_{x}$ and $G_{y}$ denotes x derivatives and y derivatives of $\tau_{1}\left(x,y\right)$, respectively. Later, the edge map was constructed using a threshold function, as follows:

(4) $$EMp\left(x,y\right)=\{\begin{array}{c} 1\;G\left(x,y\right)<T\\ 0\;G\left(x,y\right)\geq T \end{array},$$

From this equation, we considered the pixels with values higher than the threshold (T=0.55). These pixels were used for texture histogram computation (HC). Later on, α and β were calculated, where α denotes minimum and β denotes maximum pixel value. The grey levels whose value lied between α and β, were represented as HC. Finally, the cumulative distribution function (CDF) and the transfer functions were applied to obtain an enhanced image. This was defined by Equations (5) and (6), as follows:

(5) $$CDF\left(i\right)=\sum_{i=0}^{n}Th\left(i\right),$$

(6) $$F_{\tau }=\tau_{0}+\left(L-1-\tau_{0}\right)CDF,$$

The resultant image $\tau_{2}\left(x,y\right)\in CDF\left(i\right)\&F_{\tau }$ was passed to the DCT method to refine the local contrast of the tumor region. Mathematically, this was computed as follows:

(7) $$\tau_{xy}=\{\begin{array}{c} \frac{1}{\sqrt{M}}\;\;\;x=0,\;\;0\leq y\leq M-1\\ \sqrt{\frac{2}{M}\;}\;Cos\frac{\pi \left(2y+1\right)x}{2M}\;1\leq x\leq M-1 \end{array},$$

Hence, using $\tau_{xy}$, the DCT method was applied to an image $\tau_{2}\left(x,y\right)$, as follows:

(8) $$\tau_{3}\left(x,y\right)=\tau_{xy}\times \tau_{2}\times \tau_{xy},$$

**Figure 3:**
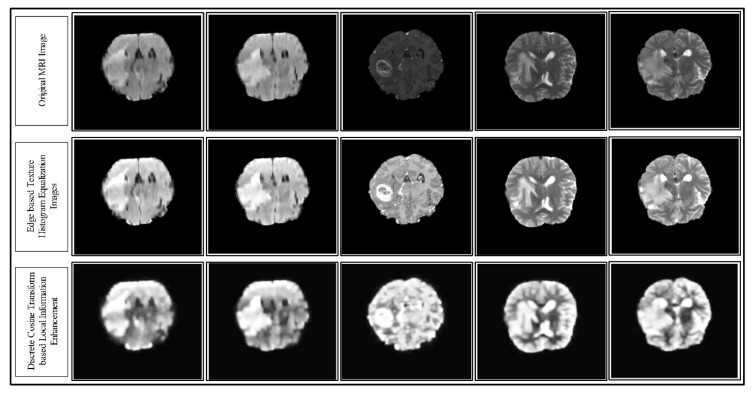
Results of the proposed hybrid local contrast enhancement using the MRI images of the Multimodal BRATS 2018 dataset. The results are shown from top to bottom.

As $\tau_{xy}$ is a real orthogonal matrix and its inverse could be computed as:

(9) $${\left[\tau_{3}\left(x,y\right)\right]}^{-1}=\tau_{xy}\times \tau_{2}\times {\left[\tau_{xy}\right]}^{t}=1,$$

where t denotes the transpose of an image. Hence, the representation of the final DCT enhanced image $\tau_{3}\left(x,y\right)$ is depicted in Figure 3. In this figure, the sample enhancement results are presented for each step (top to bottom).

### 3.2. Deep Learning Features

**Figure 4:**
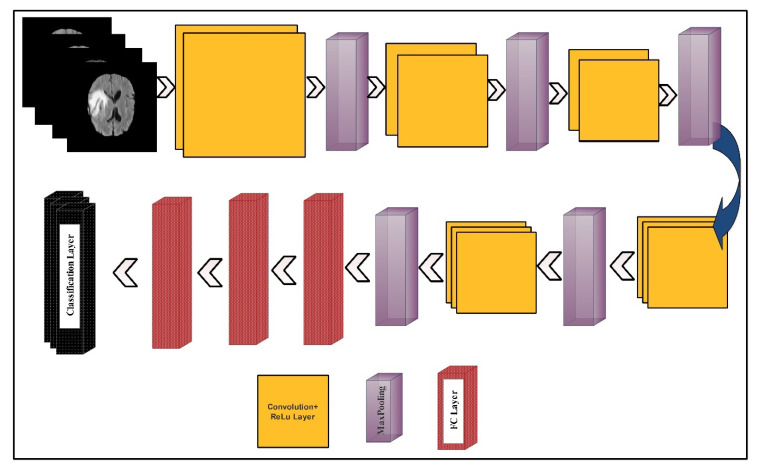
A layered wise architecture of the VGG16 deep learning model.

**Figure 5:**
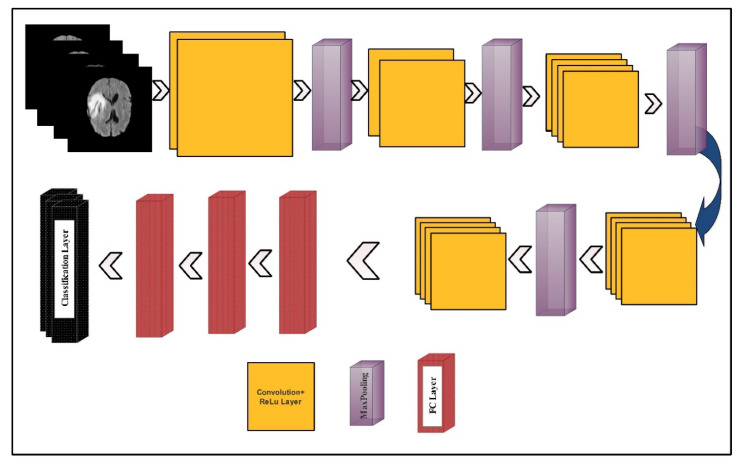
A layered wise architecture of the VGG19 deep learning model.

The deep learning features were extracted using two pre-trained deep CNN models—VGG16 and VGG19. The visual representation of both models is shown in Figure 4 and Figure 5, respectively.

The VGG16 model consisted of 12 convolution layers, 15 ReLu activation layers, five max-pooling layers, three fully connected (FC) layers, and one Softmax layer, as a classification layer. The input layer size was  224×224×3. The number of filters in the first convolution layer was 64, and the filter size was  3×3×3, along with a stride of  1×1. In the next convolution layer, the number of filters was not updated but the filter size was updated to  3×3×64. Further, the dimension of learnable weights was 3×3×64×64, which were 3×3×3×64 in the first convolution layer. The learnable weights of each convolution layer were updated according to the number of filters and the filter size. In the first max-pooling layer, a 2×2 filter size was opted along with the same stride 2×2. After the convolution layers, three FC layers were added. The learnable weights dimension of the first FC layer was  4096×25088. After a 50% dropout, the weights matrix size of the second FC layer was  4096×4096. Another dropout layer was added and a ratio of 50% was set. The resultant weight matrix used as an input of the third layer (denoted as FC8) returned a weight matrix of dimension 1000×4096. Finally, the Softmax function and the classification layers were added for the final classification.

VGG19 model consists of a series of 16 convolution layers, 19 ReLu activation layers, four max-pooling layers, three FC layers, and one Softmax layer as a classification layer. The input layer size was  224×224×3. The number of filters in the first convolution layer was 64, and the filter size was  3×3×3. This filter size was updated according to the number of filters. In the first max-pooling layer, a 2×2 filter size was opted along with the same stride. After the convolution layers, three FC layers were added. The weights dimension of the first FC layer was 4096×25088. After a 50% dropout, the weights matrix size of the second FC layer was 4096×4096. The resultant weight matrix used as an input of the third layer (denoted as FC8) returned a weight matrix of dimension  1000×4096.

### 3.3. Network Modification for Transfer Learning

Using domain adaptation transfer learning (TL) [43], we retrained both models (VGG16 and VGG19) on the BRATS datasets, without changes in any parameters. In the tuning process, first, we loaded the brain datasets and set the training/testing ratio to 60:40. Further, the labels of each image were also defined. Then, we set input and output layers for training. This process was conducted for both deep learning models. In this paper, for the VGG16 model, the input convolution layer (conv_1) was employed, where the number of filters was 64, and the filter size was  3×3×64. The selected output layer was FC8. Then, we performed activation on this layer and trained a new modified CNN network that included only the brain image features. The last two layers, namely, the classification and Softmax layers were removed. In the output, the resultant learnable weights vector length was 4×4096, and the feature-length was  1×1000. Hence, for n images, the feature vector length was N×1000, denoted by $\;\eta_{i}$. Similarly, for the VGG19 model, the last two layers were removed. The convolution layer (conv_1) was employed as an input with 64 filters, and the filter size was  3×3×64. The selected output layer was FC8, which we chose for the activation function. The activation function was performed on this layer and trained a new modified CNN network that included only the brain image features. The dimension of the learnable weight matrix was 4×4096, and the length of the extracted feature vector was  1×1000. For n brain images, the feature vector length should be N×1000 and should be denoted by $\;\eta_{j}$.

### 3.4. Feature Selection

The main motive of the feature selection step was to remove the redundancy among features and select only those features that were robust for the correct classification. The second motive of this step was to minimize the number of predictors, which helped in the fast execution of the testing process. To inspire with these two essential functionalities, we implemented a technique named correntropy via mutual learning and ELM (CML-ELM). The working of this method is presented in Algorithm 1:
**Algorithm 1** Proposed feature selection method using CML-ELM.**Input:**$\;\eta_{i}$**,**$\;\eta_{i}\in \left\{\eta_{1},\eta_{2},\;\eta_{3},\ldots ,\;\eta_{i}\right\}$**Output:**$S_{w}\left(i\right),\;S_{w}\left(i\right)\in \left\{S_{w}\left(1\right),\;S_{w}\left(2\right),\ldots ,\;S_{w}\left(i\right)\right\}$**Start****Step 1: Parameters Initialization**          $S_{w}\left(1\right)=\eta_{i},\;i=0,1,2,3,\ldots n$               $\alpha_{-1}=0$               $\alpha_{0}=1$               $LR=LR_{0}$**Step 2: For**i=1**to K****do**              $b_{i}=\frac{\alpha_{i-2}-1}{\alpha_{i-1}}$        $A^{i}=S_{w}\left(i\right)+b_{i}\left(S_{w}\left(i\right)-S_{w}\left(i-1\right)\right)$**Step 3: Update**$S_{w}\left(i+1\right)$**Step 4: Find the minimum value of**LR**among**$(LR_{\left(i-1\right)}$, $2LR_{\left(i-1\right)}$**,**$3LR_{\left(i-1\right)}$**,…)****:-**$f\left(S_{w}\left(i\right)\right)\leq g_{\left(LR_{i},\;U^{i}\right)}S_{w}\left(i+1\right)$**Step 5: Passed computed LR values in ELM classifier****Step 6: Find MSER for ELM classifier****Step 7: If MSER**≥0.1**Update**$LR_{i+1}$**Step 8:**$\alpha_{i+1}=\frac{1+\sqrt{1+4_{i}^{2}}}{2}$**End For****End**

In the above algorithm, the notation $\;\eta_{i}$ denotes the original feature vector of the VGG16 deep learning model, $S_{w}\left(i\right)$ means selected feature vector, LR denotes regularization parameter, $b_{i}$ is a selected parameter, $A^{i}$ is an affine combination of $S_{w}\left(i\right)$ and $S_{w}\left(i-1\right)$, MSER denotes mean squared error, computed by Equation (10), and the updating of features $S_{w}\left(i+1\right)$ are done by Equation (11).

(10) $$MSER=\frac{1}{n}\sum_{i=1}^{K}{\left(LR_{i}-\hat{LR_{i}}\right)}^{2},$$

(11) $$S_{w}\left(i+1\right)=\arg min\;S_{w}\frac{1}{2}{||S_{w}-V||}_{F}^{2}\frac{1}{LR_{i}}f_{ns}\left(S_{w}\right),$$

(12) $$S_{w}\left(i+1\right)=\underset{S_{w}\left(1\right),\ldots S_{w}\left(D\right)}{\mathrm{argmin}}\frac{1}{2}\sum_{j=1}^{D}{||S_{w}\left(j\right)-V_{j}||}_{2}^{2}+\frac{p_{0}}{LR_{i}}f_{ns}{||S_{w}\left(j\right)||}_{2},$$

(13) $$V=A^{i}-\frac{1}{LR_{i}\;f^{\prime }\left(A^{i}\right)},$$

where the $LR_{i}$ denotes the observed features, and $\hat{LR_{i}}$ denotes the predicted features. Each time, the MSER was calculated, and if its value was greater than or equal to 0.1, then the features were updated, iterating this process for 1000 times. If the target was not achieved, then the last iteration features were selected for the classification. Finally, a robust vector was obtained, where the dimension of this vector was $X_{1}\times K$ and was denoted by $\eta_{S_{w}}\left(1\right)$, where the K stood for the number of selected features and $X_{1}$ denoted the total number of images. This feature selection process was also performed for the VGG19 feature vector $\;\eta_{j}$ and obtained a robust feature vector of dimension $X_{2}\times K$ and denoted by $\eta_{S_{w}}\left(2\right),$ where $X_{2}$ was the number of observations, and K represented the number of selected features.

### 3.5. Feature Fusion and Classification

Finally, the selected feature vectors were fused in one matrix using the PLS-based fusion approach. Consider $\eta_{S_{w}}\left(1\right)$ and $\eta_{S_{w}}\left(2\right)$ are two selected feature vectors of dimension $X_{1}\times K$ and $X_{2}\times K$. Suppose $\eta_{S_{w}}\left(j\right)$ represents a fused vector of dimension $X_{3}\times K$. Further, we assumed that the central variables $\vec{U}$ and $\vec{V}$ were zero mean, where $\vec{U}\in \eta_{S_{w}}\left(1\right)$ and $\vec{V}\in \eta_{S_{w}}\left(2\right)$. Let $\delta_{uv}=\vec{U}\vec{V}$ and $\delta_{vu}=\delta_{uv}^{T}\left(\left(\frac{1}{n}-1\right)\delta_{uv}\right)$ represent between set covariance among vectors $\vec{U}$ and $\vec{V}$. The PLS held correlated features for fusion. Further, the fusion process through PLS also minimized the number of predictors. Mathematically, the decomposition method among $\vec{U}$ and $\vec{V}$ was defined as follows:

(14) $$\vec{U}=\sum_{i=}^{d}\eta_{i}\;\eta_{S_{w}}{\left(1i\right)}^{T}=E,$$

(15) $$\vec{V}=\sum_{i=}^{d}\eta_{i}\;\eta_{S_{w}}{\left(2i\right)}^{T}=F,$$

When using PLS, a pair of directions among $u_{i}$ and $v_{i}$ was found, as follows:

(16) $$\left\{u_{i};v_{i}\right\}=arg\underset{u^{T}u=v^{T}v=1}{\max}Cov\left({\vec{U}}^{T}u,\;{\vec{V}}^{T}v\right),$$

(17) $$\left\{u_{i};v_{i}\right\}=\underset{u^{T}u=v^{T}v=1}{\mathrm{argmax}}u^{T}\delta_{uv}\;v,\;for\;i=1,2,3,\ldots d\;a=1,$$

These pairs were combined in one matrix and a resultant vector was obtained with $X_{3}\times K$ dimension. The fused vector was represented by $\eta_{S_{w}}\left(j\right)$. Later on, this vector was passed to ELM [44] for the final classification. The formulation of ELM was given as follows. For L hidden layers node, the activation function g(x) was defined as follows:

(18) $$\sum_{i=1}^{L}\beta_{i}g_{i}\left(u_{i}\right)=\sum_{j=1}^{L}\beta_{i}g\left(u_{i}.u_{j}+B_{i}\right),$$

(19) $$\beta^{T}=O,$$

where L denotes a hidden layer, which was initialized as one in this work, $\beta_{i}$ denotes the output weight vector, $u_{i}$ is the input weight vector coming to the hidden layer, $B_{i}$ denotes the offset value, H is the output hidden layer node, $u_{i}.u_{j}$ means an inner product of $u_{i}$, and O is the expected output. Equation (19) was solved as:

(20) $${\hat{\beta }}_{ELM}=\underset{\beta }{\mathrm{argmin}}||\beta^{T}H-O||,$$

**Figure 6:**
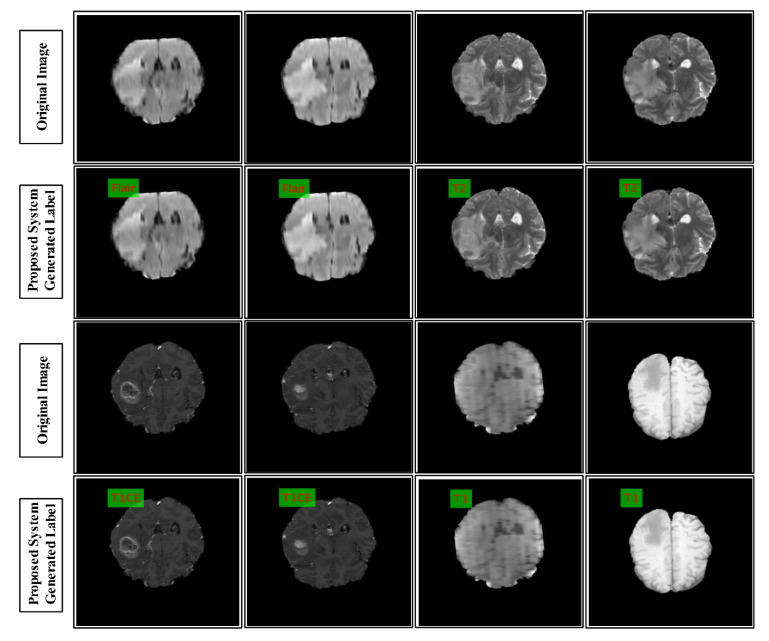
Labeled results generated by the proposed system. The label is shown in red on green square.

To further improve the stability of ELM, we defined a minimization function as:

(21) $$\underset{w}{\min}\frac{1}{2}||\beta ||+\frac{1}{2}c\sum_{i=1}^{N}{||\epsilon_{i}||}^{2}\;s.t.\;\beta^{T}h\left(u_{i}\right)=t_{i}-\epsilon_{i},$$

where $\epsilon_{i}$ denotes training error, $t_{i}$ indicates corresponding labels to the sample $u_{i},$ and c denotes the penalty parameter. The labeled results of the proposed architecture are given in Figure 6.

### 3.6. Experimental Results and Analysis

We present the classification results for the proposed ELM classifier using three datasets, namely, BraTS 2015, BraTS 2017, and BraTS 2018. For all datasets, a 60–40 split ratio was used along with 10-fold cross-validation. The results are provided for two different pipeline procedures, namely; (i) feature extraction from FC layer seven and a performed feature selection approach that followed the feature fusion and classification and (ii) which followed the proposed architecture, as given in Figure 2. For the sake of comparison, we also provided the results for four well-known classifiers, like Naïve Bayes, Multiclass Support Vector Machine (MSVM), Softmax, and Ensemble Tree, as baselines. The performance of all classifiers was validated by the following measures, namely accuracy and FNR measures. Furthermore, the clock time taken by each classifier was also reported to give the reader an idea about the classification time during the testing process. All simulations of the proposed technique were conducted on MATLAB 2019b (MathWorks, Natick, MA, USA). The personal Desktop Computer with 16 GB RAM and 128 GB SSD was used for these experiments. A graphics processing unit (GPU) was also utilized for feature extraction and classification, which significantly helped in improving the classification time. The execution time was also noted during the testing process; however, it was not consistent and was only based on the execution platform.

### 3.7. Results for the BraTS 2015 Dataset

Table 1 presents the classification results for the BraTS 2015 dataset. The results were provided for the proposed classifier, as well as the existing well-known classifiers, such as Naïve Bayes, MSVM, Softmax, and Ensemble Tree. These results were provided for two experimental pipeline procedures, as mentioned above. Apart from the validation measures in terms of accuracy and FNR, the results were also provided for the classification time in seconds. The entries in the bold represent the best results. It can be seen from Table 1 that the minimum accuracy achieved was 91.48% for Softmax. The maximum accuracy of 98.16% (FNR = 1.74%) was achieved by the ELM classifier, which used the proposed method.

The proposed selection scheme also reduced the classification time during the testing process. In Table 1, time is given for all classifiers, which clearly shows that the time for the proposed method was lesser than that compared to Pro-FC7. The classification time for Softmax was minimum (81.02 s), using the proposed method. Though the classification time for the proposed classifier was not minimum (87.41 s), it was still quite close to Softmax and considerably lower, as compared to the rest of classifiers.

The results of the proposed method on the ELM classifier were also verified by the confusion matrix values presented in Table 2. The diagonal values showed the correct classification rate of each tumor class. The maximum achieved accuracy of Pro-FC7 was 96.02% for ELM (Table 1), which could also be verified by the confusion matrix in Table 3.

### 3.8. Results for the BraTS 2017 Dataset

Table 4 presents the classification results for the BraTS 2017 dataset. Results are provided for the proposed method along with several other well-known classifiers, such as Naïve Bayes, MSVM, Softmax, and Ensemble Tree. These results were provided for two experimental pipeline procedures, as mentioned above. Apart from the validation measures in terms of accuracy and FNR, results were also provided for the classification time in seconds. It can be clearly seen from Table 4 that the ELM classifier, which used the proposed method, had an accuracy of 97.26% and an FNR of 2.74%. The minimum met accuracy was 90.09% for Softmax.

The proposed selection scheme also reduced the classification time during the testing process, as was evident from the results shown in the last column in Table 4. The classification time for ELM was minimum (89.64 sec) using the proposed method, which clearly showed the improved efficiency of the ELM classifier.

The results of the proposed method on the ELM classifier could also be verified by the confusion matrix in Table 5. The diagonal values showed the correct classification rate of each tumor class, which were 96.24%, 98.66%, 97.20%, and 97% for the T1, T1CE, T2, and Flair tumors. The maximum achieved accuracy of Pro-FC7 was 95.82% for ELM, which could be further verified by the results in Table 6.

### 3.9. Results of the BraTS 2018 Dataset

Table 7 presents the classification results for the BraTS 2018 dataset. Results were provided for the proposed method, as well as for other well-known classifiers, such as Naïve Bayes, MSVM, Softmax, and Ensemble Tree. These results were provided for two experimental pipeline procedures, as discussed earlier in Section 3. It can be seen from this table that the maximum achieved accuracy was 93.40% for the ELM classifier, using the proposed method. The noted FNR rate was 6.60%. The minimum achieved accuracy was 89.49%, using the proposed method for the Naïve Bayes classifier.

The classification accuracy was also computed for Pro-FC7 to analyze the proposed results. For Pro-FC7, the maximum achieved accuracy was 91.69% for the ELM classifier. The accuracy of ELM using the proposed method and Pro-FC7 was further verified through Table 8 and Table 9. In both these tables, the diagonal values represent the correct predicted rate of each tumor class, such as T1, T2, T1CE, and Flair.

Time was measured for each classifier during the testing process and presented in Table 7. We used tic-toc commands to compute the testing computational time of proposed method. In this table, it was observed that the best execution time was (63.83 s) for the ELM classifier, using the proposed method. However, this time was based on the platform that was used like GPU, system RAM, etc. Based on the presented results of accuracy and the testing execution time, the effectiveness of the proposed method was apparent for the accurate and efficient brain tumor type classification.

### 3.10. Results of the Contrast Enhancement

**Figure 7:**
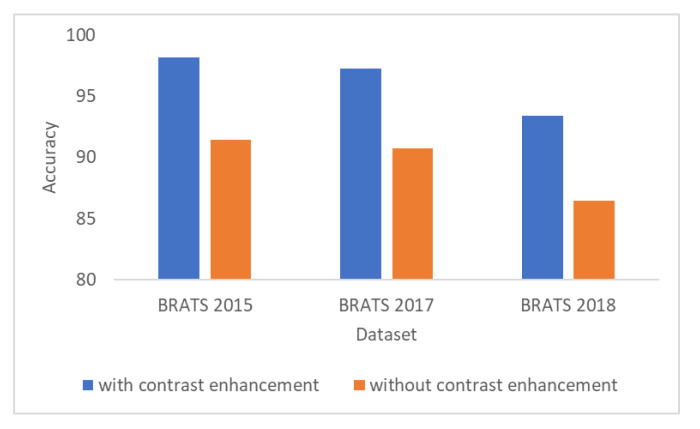
Accuracy results with and without employing the contrast enhancement step.

Figure 7 shows the importance of the contrast enhancement step. In this figure, it can be seen that, if the contrast enhancement step is not employed, the results show a decrease of almost 7% of accuracy for all BraTS datasets.

### 3.11. Statistical Analysis of Results

**Figure 8:**
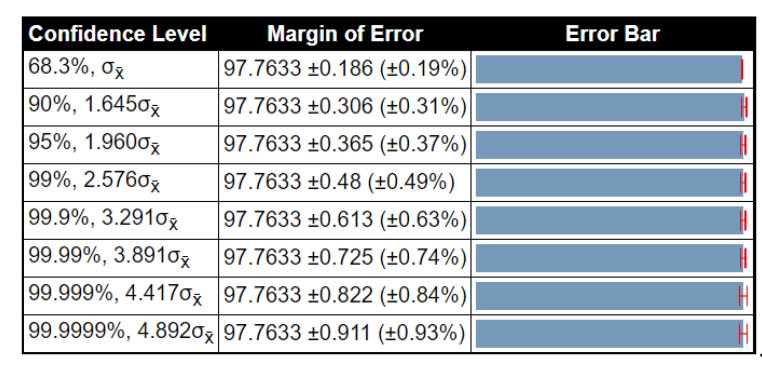
Confidence interval of statistical test for different critical values of *t*-test (BraTS 2015 dataset).

**Figure 9:**
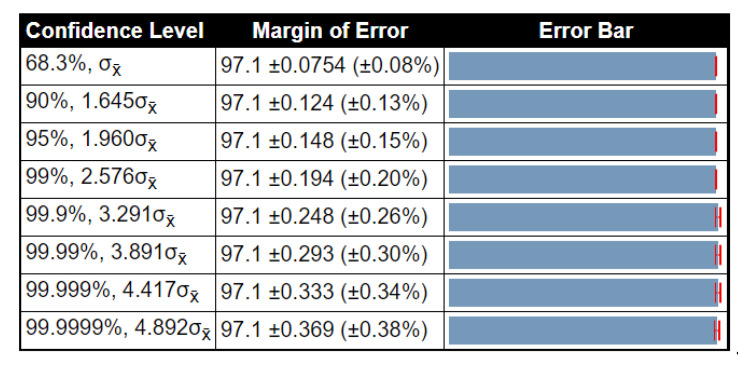
Confidence interval of statistical test for different critical value of *t*-test (BraTS 2017 dataset).

**Figure 10:**
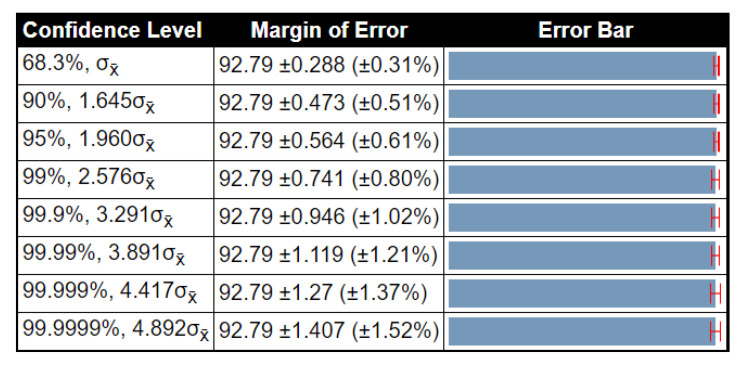
Confidence interval of statistical test for different critical value of *t*-test (BraTS 2018 dataset).

To examine the stability of the proposed method results, a detailed statistical analysis was conducted in terms of variance, standard deviation, and standard error mean (SEM). The noted values were obtained after 1000 iterations. The detailed analysis of the proposed method for the BraTs2015 dataset is given in Table 10. In this table, the accuracy of ELM had low variability, and SEM was 0.1862, which was better than that compared to other methods. Table 11 shows the detailed analysis of the proposed method using the BraTs2017 dataset. The accuracy of ELM was better than that compared to other listed classifiers (SEM is 0.0754). Table 12 illustrates the analysis results for the BraTs2018 dataset. Here, the SEM for the proposed method was 0.2875. As compared to other classifiers, it was better, and the results were stable after the selected iterations. Overall, the results of the proposed method were more stable for all listed classifiers. Moreover, we also plotted the confidence interval of ELM at different confidence levels (CL), such as 90%, 95%, 99%, etc., as shown in Figure 8, Figure 9 and Figure 10. As shown in Figure 8, at 95% CL, the margin of error was 97.763 ± 0.365 (±0.37%). Similarly, in Figure 9 and Figure 10, the margin of error at 95% CL was 97.1 ± 0.148 (±0.15%) and 92.79 ± 0.564 (±0.61%), respectively. Based on these values, it was shown that our method was significantly better than that compared to other classifiers.

## 4. Discussion

We discuss the results of the proposed method from a critical point of view. The labeled results are illustrated in Figure 6. Three BraTs datasets were used for the validation of the proposed method. The numerical results are presented in Table 1, Table 4, and Table 7. The results presented in these tables were validated through two pipeline procedures, as mentioned in Section 3. The results showed that the accuracy of Pro-FC7 was less, as compared to the proposed architecture. The main reason for the degradation of the classification accuracy was the number of features. For the architecture of VGG19, the feature length on FC7 was 4096, whereas, the feature length of FC8 was 1000; therefore, during the selection process, the target MSER could not be met. Moreover, due to higher number of features, the execution time was also higher for Pro-FC7, as compared to the proposed method.

To give the reader an idea of comparison with the existing techniques, we briefly mentioned some published results. In [24], the authors presented a deep-learning-based system and used the BraTs dataset series for the experimental process. They achieved an accuracy of 97.8%, 96.9%, and 92.5% for the BraTs2015, BraTs2017, and BraTs2018, respectively. Sajjad et al. [23] presented a deep learning model and evaluated this on two datasets—Brain tumor and Radiopaedia. They achieved an accuracy of 94.58% and 90.67%, respectively, on both. Toğaçar et al. [35] achieved an average 96.77% accuracy for the classification of healthy and tumor MRI images. The proposed method achieved an accuracy of 98.16%, 97.26%, and 93.40%, which was better than that compared to the accuracy reported for the state-of-the-art techniques. Additionally, the worst time complexity of our algorithm was $O\left(n^{3}+k\right)+C$, where k represents the number of iterations and C is a constant term.

In addition, we also calculated the Mathew correlation coefficient (MCC) measure for the ELM classifier; the results are given in Table 13. In this table, it is shown that the MCC values were closer to 1, which showed the better prediction performance of the proposed scheme.

## 5. Conclusions

This paper presents a fully automated deep learning system, along with contrast enhancement for multimodal brain tumor classification. The strength of this work was in three steps. First, in the preprocessing step, contrast stretching using edge-based texture HE was employed to increase the local contrast of the tumor region. Secondly, the selection of robust deep learning features by implementing correntropy via mutual learning and ELM (CML-ELM) was utilized. Using CML-ELM, the robust features were computed, which were fused through the PLS-based approach, in a later stage. Third, the ELM classifier was implemented for the classification of proposed tumors into the relevant category. The experimental process was conducted on the BraTs datasets and the results showed an improved accuracy (98.16%, 97.26%, and 93.40%, for the BraTs2015, BraTs2017, and BraTs2018 datasets, respectively). The feature selection process was not only helpful for improving the classification accuracy, but also resulted in the reduction of the computational time. Finally, the accuracy results of the proposed method were stable, which could be concluded on the basis of the presented results.

## Figures and Tables

**Table 1:** Classification results for the BraTS 2015 dataset.

Classifier	Feature Selection Technique	Validation Measures		
		Accuracy (%)	FNR (%)	Testing Time (s)
**Naïve Bayes**	Pro-FC7	93.29	6.71	117.68
	Proposed	94.19	5.81	104.02
**MSVM**	Pro-FC7	92.59	7.41	136.31
	Proposed	94.66	5.34	101.66
**Softmax**	Pro-FC7	91.48	8.52	96.69
	Proposed	93.98	6.02	**81.02**
**Ensemble Tree**	Pro-FC7	92.43	7.57	137.60
	Proposed	95.67	4.33	104.59
**ELM**	Pro-FC7	96.02	3.98	99.42
	Proposed	**98.16**	**1.74**	87.41

Pro-FC7 defines feature extraction from the FC7 layer and performed feature selection, as well as fusion, and ‘Proposed’ denotes the proposed classifier architecture, as given in Figure 2. The best values are shown in bold.

**Table 2:** Confusion matrix of the proposed method for the BraTS 2017 dataset (Proposed).

Class	T1	T1CE	T2	Flair
**T1**	98.42%	<1%	0%	<1%
**T1CE**	1%	96.00%	2%	1%
**T2**	0%	0%	99.46%	<1%
**Flair**	<1%	<1%	<1%	98.80%

Grey background shows accuracy rate.

**Table 3:** Confusion matrix of ELM using Pro-FC7 approach for the BraTS 2017 dataset.

Class	T1	T1CE	T2	Flair
**T1**	97.16%	<1%	2%	0%
**T1CE**	<1%	95.24%	3%	1%
**T2**	<1%	2%	97.60%	0%
**Flair**	0%	2%	3%	94.00%

Grey background shows accuracy rate.

**Table 4:** Classification results for the BraTS 2017 dataset.

Classifier	Feature Selection Technique	Validation Measure		
		Accuracy (%)	FNR (%)	Testing Time (s)
**Naïve Bayes**	Pro-FC7	91.59	8.41	197.46
	Proposed	93.66	6.34	104.59
**MSVM**	Pro-FC7	90.09	9.91	211.62
	Proposed	94.58	5.42	171.42
**Softmax**	Pro-FC7	91.67	8.33	111.44
	Proposed	93.98	6.02	91.25
**Ensemble Tree**	Pro-FC7	93.69	6.31	147.38
	Proposed	95.42	4.58	101.29
**ELM**	Pro-FC7	95.82	4.18	107.59
	**Proposed**	**97.26**	**2.74**	**89.64**

The best values are shown in bold.

**Table 5:** Confusion matrix of the proposed method for the BraTS 2017 dataset.

Class	T1	T1CE	T2	Flair
**T1**	96.24%	2%	1%	<1%
**T1CE**	<1%	98.66%	0%	1%
**T2**	2%	0%	97.20	<1%
**Flair**	1%	0%	2%	97.00%

Grey background shows accuracy rate.

**Table 6:** Confusion matrix of ELM using the Pro-FC7 approach for the BraTS 2017 dataset.

Class	T1	T1 CE	T2	Flair
**T1**	94.20%	4%	1%	<1%
**T1 CE**	4%	94.84%	3%	2%
**T2**	0%	3%	96.68%	<1%
**Flair**	<1%	1%	2%	96.02%

Grey background shows accuracy rate.

**Table 7:** Classification results for the BraTS 2018 Dataset.

Classifier	Feature Selection Technique	Validation Measure		
		Accuracy	FNR	Testing Time (s)
**Naïve Bayes**	Pro-FC7	87.63	12.37	204.31
	Proposed	89.49	10.51	117.62
**MSVM**	Pro-FC7	88.19	11.81	207.56
	Proposed	91.34	8.66	167.49
**Softmax**	Pro-FC7	90.26	9.74	131.31
	Proposed	92.42	7.58	91.63
**Ensemble Tree**	Pro-FC7	89.16	10.84	151.34
	Proposed	91.79	8.21	106.12
**ELM**	Pro-FC7	91.69	8.31	97.04
	**Proposed**	**93.40**	**6.60**	**63.83**

The best values are shown in bold.

**Table 8:** Confusion matrix of the proposed method for the BraTS 2017 dataset.

Class	T1	T1CE	T2	Flair
**T1**	89.40%	7%	<1%	3%
**T1CE**	3%	94.60%	<1%	2%
**T2**	2%	4%	93.20%	<1%
**Flair**	1%	0%	3%	96.00%

Grey background shows accuracy rate.

**Table 9:** Confusion matrix of ELM using the Pro-FC7 approach for the BraTS 2017 dataset.

Class	T1	T1CE	T2	Flair
**T1**	88.18%	6%	3%	<3%
**T1CE**	<1%	92.16%	6%	1%
**T2**	1%	7%	91.62%	<1%
**Flair**	2%	<1%	5%	92.14%

Grey background shows accuracy rate.

**Table 10:** Detailed statistical analysis of the proposed method using the BraTS 2015 dataset.

Method	Min (%)	Avg (%)	Max (%)	$\sigma^{2}$	σ	SEM
Naïve Bayes	92.47	93.33	94.9	0.493	0.7021	0.4054
MSVM	92.19	93.42	96.66	1.016	1.0083	0.5821
Softmax	91.63	92.8	93.98	0.920	0.9593	0.5539
ET	92.98	94.32	95.67	1.206	1.0981	0.6340
**ELM**	**97.37**	**97.76**	**98.16**	**0.104**	**0.3225**	**0.1862**

The best values are shown in bold. Min, Avg, and Max are the minimum, average, and maximum accuracy, respectively. SEM—standard error of mean.

**Table 11:** Detailed statistical analysis of the proposed method using the BraTS 2017 dataset.

Method	Min (%)	Avg (%)	Max (%)	$\sigma^{2}$	σ	SEM
Naïve Bayes	91.04	92.35	93.66	1.144	1.0696	0.6175
MSVM	92.67	93.62	94.58	0.608	0.7797	0.451
Softmax	90.29	92.13	93.98	2.2693	1.5064	0.8697
ET	93.16	94.29	95.42	0.8512	0.9226	0.5326
**ELM**	**96.94**	**97.1**	**97.26**	**0.017**	**0.1306**	**0.0754**

The best values are shown in bold. Min, Avg, and Max are the minimum, average, and maximum accuracy, respectively. SEM—standard error mean.

**Table 12:** Detailed statistical analysis of the proposed method using the BraTS 2018 dataset.

Method	Min (%)	Avg (%)	Max (%)	$\sigma^{2}$	σ	SEM
Naïve Bayes	87.42	88.45	89.49	0.7141	0.8450	0.4879
MSVM	86.69	90.01	91.34	1.1704	1.0818	0.6246
Softmax	91.04	91.73	92.42	0.3174	0.5633	0.3252
ET	88.64	90.21	91.79	1.6537	1.2859	0.7424
**ELM**	**92.18**	**92.79**	**93.40**	**0.2480**	**0.4980**	**0.2875**

The best values are shown in bold. Min, Avg, and Max are the minimum, average, and maximum accuracy, respectively. SEM—standard error mean.

**Table 13:** Calculation of the Mathew correlation coefficient (MCC) value for the ELM classifier.

Dataset	Pro-FC7	Proposed	MCC
BRATS2015	√		0.8690
		√	**0.8804**
BRATS2017	√		0.8523
		√	**0.8764**
BRATS2018	√		0.8036
		√	**0.8244**

The better values are shown in bold.
